# Use of CAD–CAM Bridging Mandibular Prosthesis in Osteonecrosis of the Jaw: The Experience of Our School

**DOI:** 10.3390/jcm9113516

**Published:** 2020-10-30

**Authors:** Francesco Ricotta, Salvatore Battaglia, Federico Bolognesi, Francesco Ceccariglia, Claudio Marchetti, Achille Tarsitano

**Affiliations:** Oral and Maxillo-Facial Surgery Unit, IRCCS Policlinico di Sant’Orsola, Department of Biomedical and Neuromotor Sciences (DIBINEM), University of Bologna, 40100 Bologna, Italy; salbatt89@yahoo.it (S.B.); federico.bolognesi2@gmail.com (F.B.); ceccariglia.fc@gmail.com (F.C.); claudio.marchetti@unibo.it (C.M.); achille.tarsitano2@unibo.it (A.T.)

**Keywords:** osteonecrosis, bisphosphonates, osteoradionecrosis, computer-aided design, computer-aided manufacturing, CAD–CAM, prosthesis, bridging plate, mandibular reconstruction

## Abstract

Osteonecrosis of the jaw (ONJ) is a disease that affects the jaw. It is mainly related to radiation or bisphosphonates therapy, and the symptoms and signs consist of pain, bone exposure, inflammation of the surrounding soft tissue swelling, and secondary infection or drainage. In the case of advanced disease of the mandibular area, the treatment of choice is mandibular resection and reconstruction. In the present study, we report a case series of patients affected by ONJ and treated with a customised bridging mandibular prosthesis-only technique. From 2016 to 2018, we treated five consecutive patients affected by ONJ: three patients were affected by biphosphonate-related osteonecrosis of the mandible (BRONJ) and two were affected by osteoradionecrosis of the mandible (ORNJ). Three patients needed a soft tissue free flap to permit optimal wound closure, intra- and/or extraorally. All reconstructive procedures were carried out successfully, with no major or minor microvascular complication. The average postoperative follow-up was 24.8 (range 10–41) months. Considering that microvascular bone transfer is a high-risk procedure in BRONJ patients, we can conclude that the positioning of a customised bridging mandibular prosthesis (CBMP), whether or not associated with a microvascular soft tissue transfer, is a safe technique in terms of surgical outcome and feasibility.

## 1. Introduction

**Figure 1:**
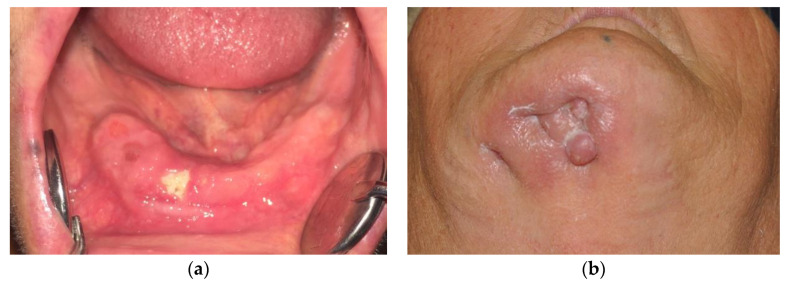
Typical signs of osteonecrosis. (**a**) Oral bone exposure; (**b**) cutaneous fistula.

Osteonecrosis of the jaw (ONJ) is a disease causing a series of symptoms such as pain, bone exposure, inflammation of the surrounding soft tissue, swelling, and secondary infection or drainage (Figure 1).

Various forms of ONJ have been described over the last few years, and a number of causes have been suggested in the literature, being frequently associated with cancer treatments (including radiation, ORNJ) and antiresorptive medications used for bone metastasis or osteoporosis (MRONJ).

Marx, in 1983, explains the pathophysiology of ORNJ using the “3H” principle (hypocellular, hypovascular, and hypoxic tissues) to describe the effect of radiation on the tissue [1] and, in 2003, was the first to describe some cases of MRONJ induced by bisphosphonates [2] (BRONJ), consequently correlated to and increasing trabecular bone density, inducing vascular insufficiency, and causing bone necrosis [3].

Decisions about treatment are based on factors such as age, sex, the stage of the disease, the severity of the BRONJ, the size and site of the lesion, exposure to drugs, and the presence of coexisting diseases [4].

In the case of advanced disease of this area, the treatment of choice is mandibular resection and reconstruction. The gold standard for mandibular reconstruction is universally recognised and consists of the replacement of the bony part of the mandible with a bony microvascular free flap supported by a reconstructive plate [5,6].

The use of computer-aided design/computer-aided manufacturing (CAD/CAM) in the case of mandibular reconstruction is crucial to allow accurate planning and affords excellent surgical outcomes, preserving vascular and nerve structures when possible [7]. This is especially true when complex 3D defects are apparent [8,9].

Although this procedure is feasible and reproducible in most patients, at times, poor oncological prognosis or poor performance status, together with other relative or absolute vascular contraindications, will force surgeons to consider other reconstructive solutions [10,11]. In these cases, the main alternative to reconstructing a mandibular defect is represented by bridging plates combined with soft tissue flaps, especially when the condyles are bilaterally preserved [12,13].

However, repairing a mandibular defect with a reconstructive plate can only lead to a series of diverse complications. The most frequent complications are rupture and oral exposure of the plate [14,15]. The latter complication is mostly due to the misplacement of the plate during the surgical reconstructive procedure. In both cases, the plate has to be removed, creating a challenging scenario for the surgeon. In fact, after the removal of a mandibular plate, a secondary approach and mandibular reconstruction are always difficult due to the lack of viable bone for the new fixation of the plate.

We have already described a pilot study for mandibular reconstruction using a customised bridging mandibular prosthesis (CBMP) without a bone-free flap [16]. To date, we have treated a total of five patients with this method.

In the present study, we report a case series of patients affected by ONJ and treated with the CBMP-only technique.

## 2. Materials and Methods

The present study is a prospective single-centre pilot study. The study was approved by the S. Orsola Hospital Ethics Committee (approval no. 57/2011/O/Disp). Between April 2016 and February 2018, 5 patients affected by ONJ were included.

The inclusion criteria were:

- Osteonecrosis of the mandible correlated to locoregional radiotherapy or induced by biphosphonates;
- Clinical and radiological signs of mandibular involvement under the course of the mandibular canal;
- Contraindications to the use of reconstruction with bone-free flap transfer;
- Reconstruction with CBMP.

The exclusion criteria were:

- Clinical contraindications to surgery;
- Reconstruction with a standard osteosynthesis plate, with or without bone-free flaps.

All surgical and reconstructive procedures were virtually planned and performed according to our CAD–CAM protocol [17].

The virtual planning began with the acquisition of a high-resolution CT scan of the craniofacial region.

Digital Imaging and Communications in Medicine (DICOM) format data were processed using the simulation software. Using this software, it was possible to create 3D virtual models of the maxillofacial skeleton. The software allowed the surgeon to plan mandibular osteotomies. Mandibular-cutting polyamide guides were used to reproduce the osteotomy site, as virtually planned. Reconstructive titanium plates were manufactured by a direct metal laser sintering method: a metal powder was fused into a solid component and melted locally using a focused laser beam. The solid-to-layer files of the guide and plate were then manufactured by direct metal laser sintering (SINTAC s.r.l., Biomedical Engineering, Trento, Italy) using an EOSINT M280 system (Electro-Optical Systems, GmbH, Munich, Germany).

### 2.1. Protocol for Computer-Assisted Design and Manufacturing Bridging Plate

**Figure 2:**
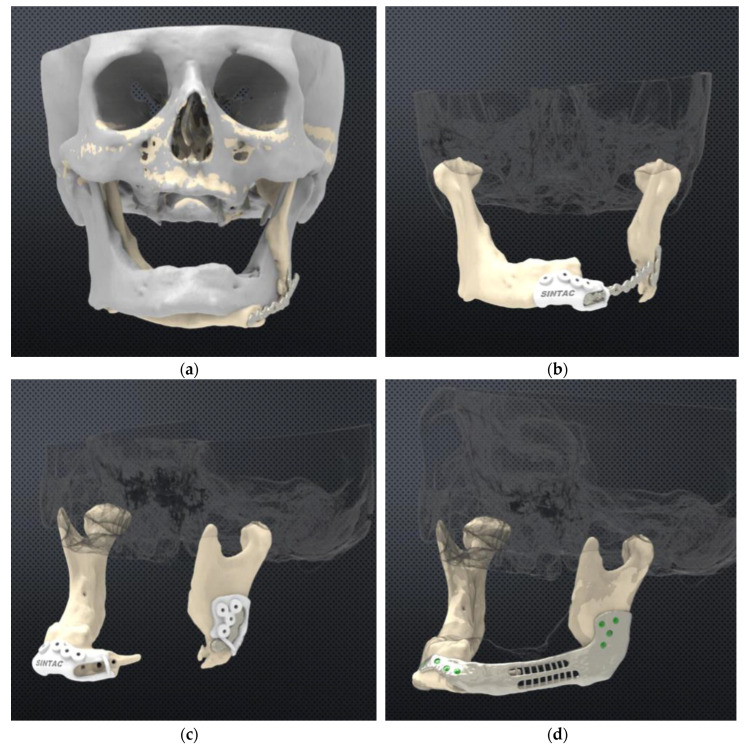
Computer-aided design of plate and drilling guides. (**a**) Superimposition of the native mandible (grey) and postresection (yellow) CT-scan data, showing mandibular displacement for plate rupture. (**b**) After mandibular segment 3d-repositioning; the design of the symphysis cutting and drilling guide, fixed on a fractured plate. (**c**) Design of ramus cutting and drilling guide. (**d**) Design of customised bridging mandibular prosthesis (CBMP).

DICOM files were processed using MIMICS software (Materialise, Leuven, Belgium) to obtain 3D virtual models, and the surgeons then engaged in virtual surgical planning (VSP). Cutting guides and CBMP were designed using 3MATIC software (Materialise). The virtual planning files were validated by surgeons (aided by a dedicated engineer) with significant experience in CAD–CAM reconstructive technique [18] (Figure 2). The CBMP model was created by reproducing the physiological conformation of the resected mandibular segment as a mandible-like bridging prosthesis, with a rounded, three-dimensional surface in order to reduce the damage at the interface between the soft tissues and the reconstructive prosthesis. The height of the CBMP was 1 cm, and the minimum thickness was 3 mm. Two retention titanium structures were designed on each osteotomy side of the plate to increase hardware stability.

Each osteotomy cutting and drilling polyamide guide considered the position of the osteotomies and the holes for the placement of plate fixation screws. The anatomical fitting of the guide on the patient’s mandible is obtained from the shape of the guide itself. If necessary, one or two customised flanges embrace the distal part of the mandible, not too distant from the osteotomy area.

The virtual planning files were verified using MAGICS software (Materialise). STL files were then used for plate manufacture (Sintac srl, Trento, Italy) via direct metal laser sintering (EOSINT M280 system; Electro-Optical Systems GmbH, Krailling, Germany), layer by layer [19]. Similarly, the STL files of the cutting and drilling guides were printed using the SLS FORMIGA P110 system (Electro-Optical Systems GmbH).

Once printed, the cutting/drilling polyamide guide and the titanium plate were cleaned using sandblasting, compressed air and, if necessary, an ultrasonic machine.

Both the cutting/drilling guides and titanium plates were then steamed in an autoclave at 132–135 °C for 60 min.

The average cost of the cutting/drilling guides was about EUR400 for each one; for CBMP, it was about EUR2200 for each one.

### 2.2. Surgical Procedure

Antibiotic prophylaxis, with amoxicillin + clavulanate acid (2 g) and clindamycin (600 mg), was performed for all patients.

**Figure 3:**
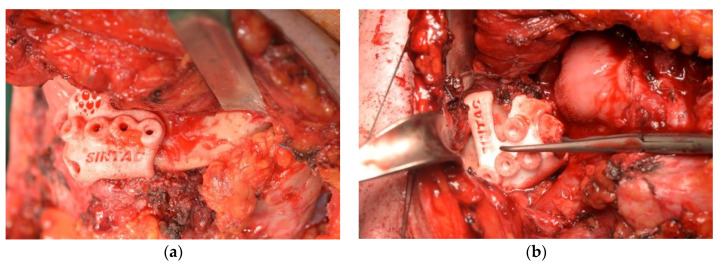
Positioning of cutting and drilling guide. (**a**) ramus positioning; (**b**) symphysis positioning.

The procedure featured the creation of surgical access and the exposure of the mandible to the extent that permitted the positioning of the cutting guides. Oral access was avoided, when possible, in order to reduce the risk of further future plate exposure (Figure 3).

To intraoperatively reproduce the planned resection, mandibular cutting and drilling guides were positioned, as virtually planned, thanks to their perfect fit on the bone surface.

In the case of primary reconstruction, once an optimal placement of the guides that were fixed by screws was obtained, osteotomies were performed with a piezosurgical instrument (Piezosurgery Medical, Mectron, Genoa, Italy).

**Figure 4:**
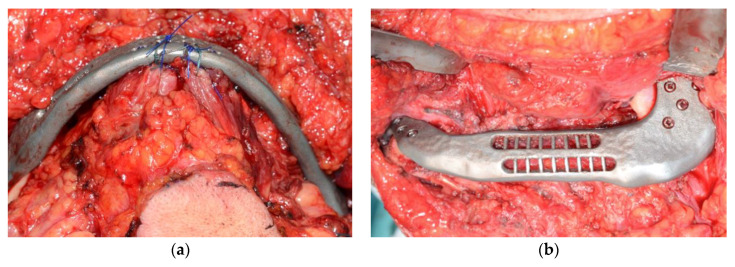
Intraoperative images of customised bridging mandibular prosthesis (CBMP) positioning. (**a**) CBMP for a lateral defect. (**b**) Suprahyoid muscle anchorage to the CBMP in an anterior defect.

After the mandibular resection was performed, the guides were removed, the CBMP was placed and fixed on the created holes, thanks to the cutting guides, using the principle of transferring holes positioning (Figure 4).

In the case of secondary reconstruction, drilling guides were positioned on the preplanned sites and fixed with screws. If a broken or misplaced plate was present, it was removed and substituted with the CBMP.

Suprahyoid muscles were anchored to the CBMP using nonresorbable sutures.

When necessary, the soft tissue free flap was harvested in order to permit an optimal wound closure, intra- and/or extraorally.

At the end of the surgery, a gastric-nose tube was positioned to help the patient’s postoperative alimentation and, when present, to maintain and clean the intraoral wound.

### 2.3. Postoperative Care

We prescribed postoperative antibiotic therapy, with amoxicilline + clavulanate acid (1 g × 3/die for 15 days) and clindamycin (600 mg × 4/die for 10 days), painkillers, and steroids.

**Figure 5:**
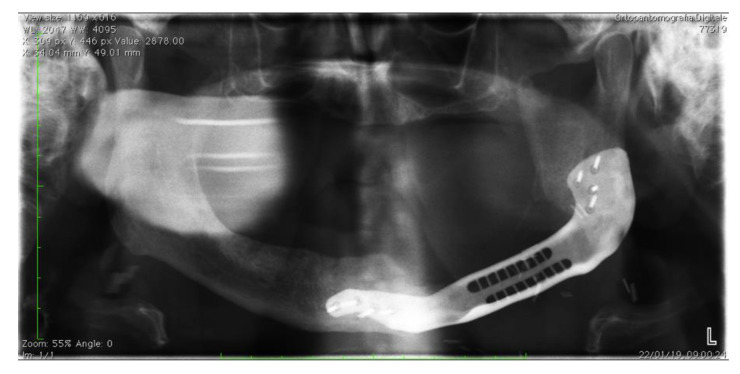
Postoperative orthopantomography in a patient treated for left-body biphosphonate-related osteonecrosis of the mandible (BRONJ)**.**

A postoperative orthopantomography was performed after the removal of the nasogastric tube (Figure 5).

**Figure 6:**
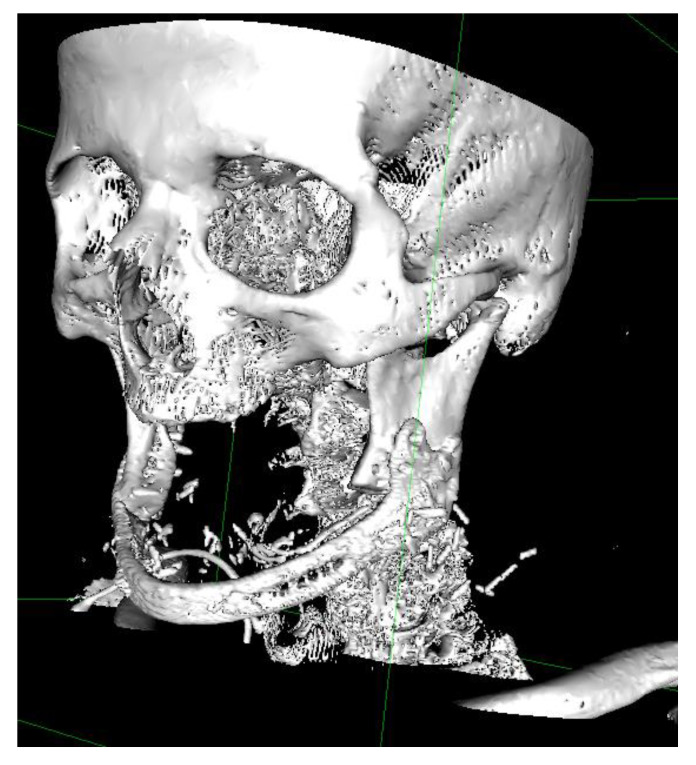
Postoperative CT-scan 3D reconstruction in a patient treated for symphysis BRONJ.

A computer tomography without contrast enhancement was performed after one month (Figure 6).

## 3. Results

From 2016 to 2018, we treated five consecutive patients affected by ONJ: three patients were affected by BRONJ and two were affected by ORNJ.

Three patients were treated with a CBMP after the rupture or misplacement of a standard plate; two patients were treated as a result of previous surgical treatment without the positioning of a plate.

In all cases, our surgery was secondary.

According to Tarsitano et al.’s classification [17], we performed three class I resections, one class II resection and one class III resection with condylar disarticulation.

Three patients needed a soft tissue free flap to permit optimal wound closure, intra- and/or extraorally. In all cases that needed a microvascular soft tissue flap, the flap harvested was an anterolateral-thigh (ALT) flap. All reconstructive procedures were carried out successfully. No major or minor microvascular complication, according to Classen and Ward’s classification [20], occurred.

The average postoperative follow-up was 24.8 (range 10–41) months.

**Figure 7:**
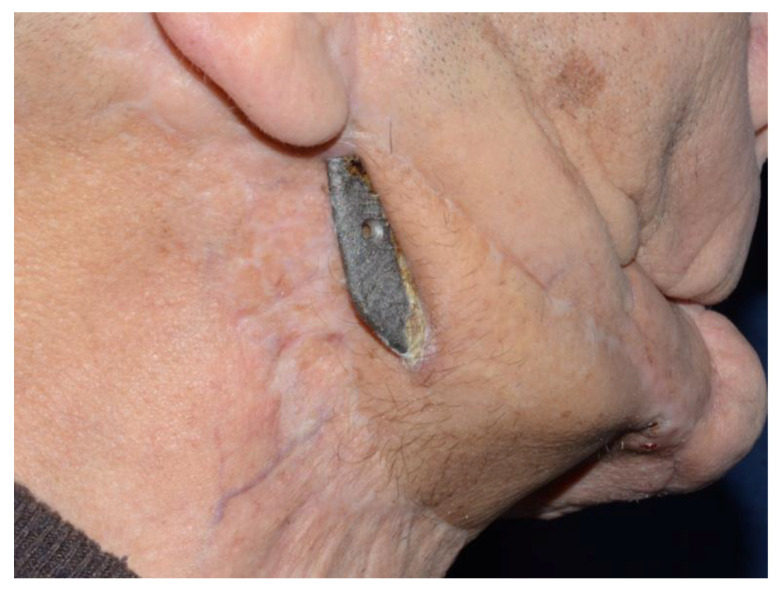
CBMP exposure in a case of ORNJ.

All patients except one (Figure 7) were alive and without exposure of the CBMP at the time of writing.

CBMP removal, after 10 months of our surgery, was performed in a single case to treat the complication that occurred.

All clinical features are summarised in Table 1.

## 4. Discussion

Nowadays, ONJ is a growing issue related to the increased life expectancy of oncologic patients.

The symptoms and signs, which most commonly affect the jaw, are pain, bone exposure, inflammation of the surrounding soft tissue, swelling, and secondary infection or drainage.

The correlation between ONJ and adjuvant therapies for cancer disease and antiresorptive medications is well described in the existing literature.

In fact, in 1983, Marx had already explained the pathophysiology of ORNJ, using the “3H” principle (hypocellular, hypovascular, and hypoxic tissues) to describe the effect of radiation on the tissue [1], and, in 2003, was the first to describe some cases of MRONJ induced by bisphosphonates [2].

The indications for the treatment depend on various factors.

Age, sex, the stage of the disease, the severity of the BRONJ, the size and site of the lesion, exposure to drugs, and the presence of coexisting diseases influence the choice of timing of surgical treatment [4].

It is clear that the clinical evaluation of the disease is not sufficient to establish the correct surgical indication. For this reason, the use of diagnostic imaging, such as computer tomography (CT) scan or magnetic resonance (MRI) is recommended for an optimal assessment of the bony involvement.

According to the cited literature, in our experience, the most important imaging feature that influences the surgical choice of mandibular resection is represented by the involvement of the mandibular canal. In particular, if there is any radiological sign of extension of the ONJ below the mandibular canal, performing a mandibular resection is mandatory.

There is a worldwide consensus that the gold standard for mandibular reconstruction consists of the replacement of the bony part of the resected mandible with a bony microvascular free flap supported by a reconstructive plate [5,6].

However, severe prognosis or poor performance status, together with other relative or absolute vascular contraindications, will force surgeons to consider other reconstructive solutions [10,11]. The main alternative to reconstructing a mandibular defect is represented by bridging plates combined with soft tissue flaps, especially when the condyles are bilaterally preserved [12,13]. In this case, the use of CAD/CAM for mandibular reconstruction is crucial to allow accurate planning and affords excellent surgical outcomes [8,9] because a CAD–CAM reconstructive plate helps to avoid the rupture of the plate itself.

In fact, bending a stock plate weakens the structure of the prosthesis and exposes the patient to a higher rate of failure of the procedure. Free flap harvesting is recommended in class II and III defects, where the loss of soft tissues has to be restored to avoid further prosthesis exposure.

In our past work, we described a pilot study for mandibular reconstruction using a customised bridging mandibular prosthesis (CBMP) without a bone-free flap for a class II defect [16]. In this paper, we present a total of five patients treated with this method. Two of these patients presented a rupture, and one of these had the misplacement of a previously positioned stock plate. The last two patients were treated in other centres without the placement of a plate, so we decided to use the CBMP to obtain an optimal repositioning of the residual mandibular segment. In these cases, the local clinical conditions and the quality of the remaining bone are usually not adequate to perform a new plate fixation. Printing a titanium CAD/CAM prosthesis helped us to have a larger anchorage surface on the bone and more bone contact.

All patients except one (Figure 6) were alive and without exposure of the CBMP at the time of writing. The single case with plate exposure was a patient with an important cutaneous retraction due to the radiotherapy and scars of previous surgery. For this reason, an ALT free flap was performed, but probably the quantity and quality of tissue was not satisfactory.

The literature has reported plate exposure as the most frequent complication. The possible factors involved are both scar contracture and a tenuous vascular supply of soft tissues overlying the plate. Consequently, the plate continues to exercise pressure on the overlying tissues and results in necrosis and exposure over time [21]. Furthermore, the disconnection of the masticatory muscles can be the cause of intraoral plate exposure, especially in patients who have undergone anterior mandibulectomy [12], such as in class II or class III defects. In these cases, there is the possibility of designing a CBMP positioned along the lingual cortex of the native mandibular bone, associated with the reconstruction of mucosal minus, with a soft tissue free flap to reduce tension and the risk of plate exposure.

Potential disadvantages of this technique include the cost of designing and prototyping the device [22], besides the fact that it is impossible to obtain implant-supported dental rehabilitation.

However, the effective cost of a CBMP should be evaluated by considering the lowering of surgical and hospitalisation time, in addition to the reduction of the need for secondary or tertiary revision procedures for plate dislocation or exposure [23].

In our described and selected cases, the part of the resected mandible was largely edentulous before surgery; therefore, the functional impairment was negligible.

## 5. Conclusions

Considering that microvascular bone transfer is a high-risk procedure in BRONJ patients, we can conclude that the positioning of a customised bridging mandibular prosthesis (CBMP), whether or not associated to a microvascular soft tissue transfer, is a safe technique in terms of surgical outcome and feasibility.

## Figures and Tables

**Table 1:** Patient characteristics overview.

Patient	Disease	Localization	Age	Type of Resection	Reconstruction	Follow-up (months)	Complications
1	ORNJ (plate rupture)	Right mandible	80	I	CBMP	22	/
2	BRONJ	Right mandible	43	I	CBMP + ALT	26	/
3	BRONJ (plate rupture)	Left mandible	74	I	CBMP	25	/
4	BRONJ (plate misplacement)	Symphysis	66	II	CBMP + ALT	41	/
5	ORNJ	Right mandible + Symphysis	69	III	CBMP(C) + ALT	10	plate exposure

ORNJ = osteoradionecrosis of the jaw; BRONJ = bisphosphonates related osteonecrosis of the jaw; CBMP = customised bridging mandibular prosthesis; ALT = anterolateral thigh free flap.

## References

[1] Marx R.E. (1983). A new concept in the treatment of osteoradionecrosis. J. Oral Maxillofac. Surg..

[2] Marx R.E. (2003). Pamidronate (Aredia) and zoledronate (Zometa) induced avascular necrosis of the jaws: A growing epidemic. J. Oral Maxillofac. Surg..

[3] Durie B.G., Katz M., Crowley J. (2005). Osteonecrosis of the jaw and bisphosphonates. N. Engl. J. Med..

[4] Yoshiga D., Nakamichi I., Yamashita Y., Yamamoto N., Yamauchi K., Nogami S., Kaneuji T., Mitsugi S., Tanaka K., Kataoka Y. (2014). Prognosis factors in the treatment of bisphosphonate-related osteonecrosis of the jaw-prognostic factors in the treatment of BRONJ. J. Clin. Exp. Dent..

[5] Lonie S., Herle P., Paddle A., Pradhan N., Birch T., Shayan R. (2016). Mandibular reconstruction: Meta-analysis of iliac versus fibula free flaps. ANZ J. Surg..

[6] Kokosis G., Schmitz R., Powers D.B., Erdmann D. (2016). Mandibular reconstruction using the free vascularized fibula graft: An overview of different modifications. Arch. Plast. Surg..

[7] Ricotta F., Battaglia S., Sandi A., Pizzigallo A., Marchetti C., Tarsitano A. (2019). Use of a CAD-CAM inferior alveolar nerve salvage template during mandibular resection for benign lesions. Acta Otorhinolaryngol. Ital..

[8] Bolognesi F., Tarsitano A., Cicciù M., Marchetti C. (2020). Surgical Management of Primary Chronic Osteomyelitis of the Jaws: The Use of Computer-Aided-Design/Computer-Aided Manufacturing Technology for Segmental Mandibular Resection. J. Craniofac. Surg..

[9] Fernández-Ferro M., Fernández-Sanromán J., Costas-López A., López-Betancourt A. (2018). Fibrous condylar dysplasia: Resection and reconstruction with a custom-made TMJ prosthesis using virtual surgical planning. J. Stomatol. Oral Maxillofac. Surg..

[10] Poli T., Ferrari S., Bianchi B., Sesenna E. (2003). Primary oromandibular reconstruction using free flaps and thorp plates in cancer patients: A 5-year experience. Head Neck.

[11] Bedogni A., Bettini G., Ferronato G., Fusetti S. (2014). Replacement of fractured reconstruction plate with customized mandible implant: A novel technique. Laryngoscope.

[12] Fanzio P.M., Chang K.P., Chen H.H., Hsu H.H., Gorantla V., Solari M.G., Kao H.K. (2015). Plate exposure after anterolateral thigh free-flap reconstruction in head and neck cancer patients with composite mandibular defects. Ann. Surg. Oncol..

[13] Kudo K., Shoji M., Yokota M., Fujioka Y. (1992). Evaluation of mandibular reconstruction techniques following resection of malignant tumors in the oral region. J. Oral Maxillofac. Surg..

[14] Tsuchiya S., Nakatsuka T., Sakuraba M., Kimata Y., Sakurai H., Nakagawa M., Takushima A. (2013). Clinical factors associated with postoperative complications and the functional outcome in mandibular reconstruction. Microsurgery.

[15] Zavattero E., Fasolis M., Garzino-Demo P., Berrone S., Ramieri G.A. (2014). Evaluation of plate-related complications and efficacy in fibula free flap mandibular reconstruction. J. Craniofac. Surg..

[16] Tarsitano A., Battaglia S., Sandi A., Marchetti C. (2017). Design of a customised bridging mandibular prosthesis for complex reconstruction: A pilot study. Acta Otorhinolaryngol. Ital..

[17] Tarsitano A., Del Corso G., Ciocca L., Scotti R., Marchetti C. (2015). Mandibular reconstructions using computer-aided design/computer-aided manufacturing: A systematic review of a defect-based reconstructive algorithm. J. Craniomaxillofac. Surg..

[18] Tarsitano A., Battaglia S., Ricotta F., Bortolani B., Cercenelli L., Marcelli E., Cipriani R., Marchetti C. (2018). Accuracy of CAD/CAM mandibular reconstruction: A three-dimensional, fully virtual outcome evaluation method. J. Craniomaxillofac. Surg..

[19] Leiggener C., Messo E., Thor A., Zeilhofer H.-F., Hirsch J.-M. (2009). A selective laser sintering guide for transferring a virtual plan to real time surgery in composite mandibular reconstruction with free fibula osseous flaps. Int. J. Oral Maxillofac. Surg..

[20] Classen D.A., Ward H. (2006). Complications in a consecutive series of 250 free flap operations. Ann. Plast. Surg..

[21] Okura M., Isomura E.T., Lida S., Kogo M. (2005). Long-term outcome and factors influencing bridging plates for mandibular reconstruction. Oral Oncol..

[22] Burton H.E., Peel S., Eggbeer D. (2018). Reporting fidelity in the literature for computer aided design and additive manufacture of implants and guides. Addit. Manuf..

[23] Tarsitano A., Battaglia S., Crimi S., Ciocca L., Scotti R., Marchetti C. (2016). Is a computer-assisted design and computer-assisted manufacturing method for mandibular reconstruction economically viable?. J. Craniomaxillofac. Surg..

